# Monosodium urate crystal-induced pro-interleukin-1β production is post-transcriptionally regulated via the p38 signaling pathway in human monocytes

**DOI:** 10.1038/srep34533

**Published:** 2016-10-03

**Authors:** Yeon-Ho Chung, Dong-Hyun Kim, Won-Woo Lee

**Affiliations:** 1 Department of Biomedical Sciences, and BK21Plus Biomedical Science Project, Seoul National University College of Medicine, 103 Daehak-ro, Jongno-gu, Seoul 110-799, South Korea; 2 Department of Microbiology and Immunology, Seoul National University College of Medicine, 103 Daehak-ro, Jongno-gu, Seoul 110-799, South Korea; 3 Cancer Research Institute, Ischemic/Hypoxic Disease Institute, and Institute of Infectious Diseases, Seoul National University College of Medicine; Seoul National University Hospital Biomedical Research Institute, 103 Daehak-ro, Jongno-gu, Seoul 110-799, South Korea

## Abstract

IL-1β is a key mediator of sterile inflammation in response to endogenous particulates, a type of damage-associated molecular pattern (DAMPs) molecule derived from damaged cells. Despite the well-known role of sterile particulates such as monosodium urate (MSU) crystals as inflammasome inducers in monocytes/macrophages, little is known regarding how pro-IL-1β synthesis is induced under sterile inflammatory conditions. We provide evidence that MSU crystals post-transcriptionally induce the rapid production of pro-IL-1β in human primary monocytes. Metabolic labeling and pull-down assays for newly-synthesized proteins clearly showed that MSU crystals rapidly, within 30 min, induce the synthesis of pro-IL-1β as well as global proteins. Notably, MSU crystal-induced pro-IL-1β synthesis is selectively dependent on the p38 MAPK pathway, whereas global protein synthesis is mediated via the mTOR, ERK1/2, and p38 pathways. Furthermore, inhibition of Mnk1, a substrate of p38, blocked MSU crystal-induced pro-IL-1β synthesis downstream of eIF4E phosphorylation. In addition, the p38 MAPK pathway leading to phosphorylation of MK2 was also critical for stabilization of pro-IL-1β mRNA following MSU stimulation. Our findings demonstrate that post-transcriptional regulation via p38 MAPK plays a central role in the rapid synthesis of pro-IL-1β in response to MSU crystals, which is an essential step for IL-1β production in human monocytes.

Inflammation is an essential part of the immune response, which is aimed at removing harmful stimuli and maintaining host tissue integrity^1^,^2^. Innate immune cells play a pivotal role for launching inflammatory responses after sensing infectious pathogens or tissue injury through a variety of pattern recognition receptors (PPRs)^3^,^4^,^5^. It is now evident that sterile inflammation, which is caused by damage-associated molecular pattern (DAMP) molecules endogenously derived from host tissue injury and necrotic cells, largely contributes to the pathogenesis of numerous acute and chronic inflammatory diseases including gout, atherosclerosis, cancer, and Alzheimer’s disease^1^,^6^. The cellular and molecular mechanisms governing sterile inflammation remain poorly defined; however IL-1 has been suggested as a central mediator of this process^1^,^6^,^7^,^8^.

IL-1β is a potent, multifunctional proinflammatory cytokine, which is primarily produced by monocytes/macrophages and is involved in a variety of immunological functions such as proliferation, activation, and differentiation as well as in the recruitment of additional inflammatory cells. Given that unchecked or prolonged production of IL-1β is implicated in various inflammatory disorders^9^,^10^,^11^, it is not surprising that its activity is tightly controlled at the transcription, translation, maturation, and secretion levels. Unlike other cytokines, IL-1β is initially produced as a bioinactive precursor (pro-IL-1β) during a *priming step* and requires subsequent intracellular proteolytic cleavage by caspase 1 for maturation. The activation of caspase 1 occurs following the assembly of an inflammasome complex during the *activation step* and leads to cleavage of pro-IL-1β to mature IL-1β, which is then released into the extracellular environment^12^.

Among a variety of DAMPs, sterile particulates including monosodium urate (MSU) and cholesterol crystals are capable of inducing robust inflammatory responses. This excessive and unremitting inflammation causes damage to healthy tissue and underlies the pathogenesis of many crystal-based diseases^13^,^14^. MSU crystals are the crystallized form of uric acid, which is the end product of purine metabolism. Upon injury cells may leak uric acid, which subsequently functions as a danger signal or DAMP which can act as an adjuvant signal in the immune system^8^,^15^. The aberrant deposition of needle-shape MSU crystals in joints or tissues causes gout, the prototypic crystal-induced cause of acute inflammation^8^,^16^. An intensively studied mechanism underling crystal-induced inflammation is the activation of the cytosolic NALP3 inflammasome by MSU crystals in monocytes/macrophages. The inflammasome is an essential signaling complex for active IL-1β production^17^,^18^,^19^, and IL-1β-driven inflammation might contribute to the development of other comorbidities including hypertension, diabetes mellitus and cardiovascular disease in patients with gout^8^,^20^.

Although both the priming and activation steps are prerequisite for productive IL-1β generation^21^, much attention has been paid to understanding how endocytosed sterile particulates initiate the activation of the NLRP3 inflammasome resulting in the production of mature IL-1β in monocytes/macrophages. Thus, a majority of studies have utilized THP-1 cells pre-stimulated with PMA, a sufficient priming stimuli for producing pro-IL-1β; however, there is accumulating evidence that the caspase-1/IL-1β activation pathway differs between primary human monocytes and the monocyte-like leukemia cell line, THP-1, and murine macrophages, cells often used to study crystal-mediated sterile inflammation^22^,^23^. For example, primary human monocytes are capable of producing and releasing IL-1β with only TLR or cytokine stimulation as the priming step due to constitutive expression of active caspase 1. In contrast, secretion of IL-1β in THP-1 cells and murine macrophages is largely dependent on both the priming and activation steps, requiring bacterial products or Phorbol 12-Myristate 13-Acetate (PMA) stimulation prior to MSU stimulation^19^.

Given that cytokines are central initiators of innate immune responses, the rapid and robust production of proinflammatory cytokines such as TNF-α is important for successful innate immunity. In order to mediate rapid and dynamic responses to stimuli and external stress, immune effector cells rely on enhancement of the stability and of translation rate of pre-existing mRNAs as an important regulatory step^24^. Recently the biosynthesis of TNF-α was shown to be controlled by protein kinases at various post-transcriptional levels^24^,^25^,^26^. Moreover, LPS-induced activation of the p38 signaling pathway is also important for IL-1β expression transcriptionally and post-transcriptionally^27^,^28^. However, little is known regarding whether sterile crystal-mediated IL-1β production is regulated through post-transcriptional mechanisms in primary human monocytes.

In activated innate immune cells only the effects of MSU crystals as inflammasome inducers have been investigated thus far, and the influence of this agonist on post-transcriptional regulation of pro-IL1β synthesis remains unclear. Thus, we hypothesized that sterile particulates, including MSU, might be involved in the production of pro-IL-1β during the priming step, which is critical for the production of active IL-1β by human monocytes.

In the present study, we demonstrate that MSU crystals induce rapid pro-IL-1β synthesis in a translation-dependent manner through the p38/Mnk1/eIF4E pathway, and that this is an essential step for IL-1β production in primary human monocytes.

## Results

### MSU crystals induce rapid production of pro-IL-1β protein without affecting its mRNA levels in primary human monocytes

**Figure 1:**
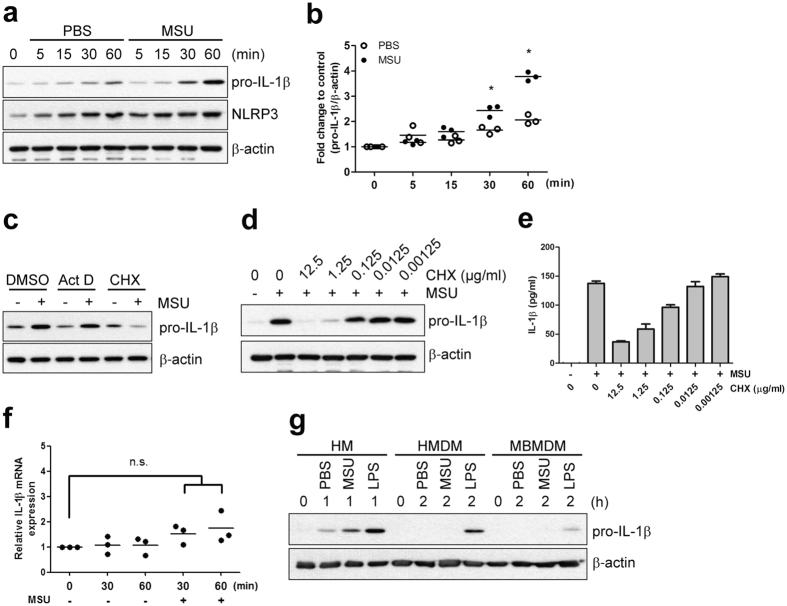
MSU crystals stimulate rapid production of pro-IL-1β protein without affecting its mRNA level in human monocytes. Human primary monocytes, freshly isolated from PBMCs of healthy donors, were stimulated with MSU crystals (400 μg/ml) or PBS as vehicle control for the indicated times. (**a**) Cell lysates were prepared at 5, 15, 30, 60 min after stimulation and the expression levels of intracellular pro-IL-1β and NLRP3 protein were analyzed by immunoblot analysis. (**b**) MSU-mediated pro-IL-1β induction is presented as the fold change compared with 0 min (before stimulation). Values were normalized to β-actin. The scatter plots show the mean of three independent experiments with three different donors. (**c**) Human monocytes were treated with MSU crystals in the presence of DMSO (vehicle control), Cyclohexamide (CHX; 0.5 μg/ml) or Actinomycin D (Act D; 2.5 μg/ml) for 60 min. The cells were harvested and pro-IL-1β was analyzed in whole cell lysates by immunoblot. Data is representative of two independent experiments with two different donors. (**d**) Human monocytes were stimulated with MSU crystals in the absence or presence of the indicated concentrations of CHX for 60 min. Cell lysates were analyzed for pro-IL-1β by immunoblot. Data is representative of two independent experiments. (**e**) The amount of IL-1β in the supernatants harvested from the culture in (**d**) was quantified by a conventional ELISA. Data is representative of two independent experiments with two different donors. Bar graph shows the mean ± SEM in triplicate. (**f**) IL-1β mRNA was quantified by real-time RT-PCR. The scatter plots show the mean of three independent experiments with three different donors. (**g**) Cell lysates were prepared from human monocytes (HM), human monocyte-derived macrophages (HMDM), and mouse bone marrow-derived macrophages (MBMDM) treated with MSU crystal (400 μg/ml), LPS (100 ng/ml), or PBS as vehicle control at the indicated times after stimulation. The expression of intracellular pro-IL-1β protein was analyzed by immunoblot analysis. Blots are representative of two independent experiments with two different donors. Data is representative of three independent experiments. *p < 0.05 and N.S., not significant by paired *t*-test in (**b**,**f**).

It has been demonstrated that IL-1β production in primary human monocytes relies on the synthesis of pro-IL-1β during an initial priming step^29^. Since the effect of sterile particulates such as MSU on IL-1β production has largely been investigated using THP-1 cells pre-stimulated with PMA, the priming stimuli for pro-IL-1β production has yet to be identified^30^. Thus, we sought to explore a possible role of MSU crystals as the priming stimuli. We first monitored the kinetics of pro-IL-1β synthesis in primary human monocytes stimulated with MSU crystals. The level of pro-IL-1β protein (p35) in the cell lysates was increased as soon as 30 min post-stimulation and the secretion of active IL-1β (p17) was detected at 60 min post-stimulation (Fig. 1a and Fig. S1). The level of pro-IL-1β protein significantly increased (2.4-fold and 3.8-fold at 30 and 60 min post stimulation, respectively) compared to the PBS-treated control (*p* < 0.05 and *p* < 0.05, respectively; Fig. 1b). This effect led to increased secretion of IL-1β by monocytes stimulated with MSU crystals at later time points (Fig. S2). This rapid production of pro-IL-1β protein was confirmed by intracellular flow cytometric analysis. Among three different monocyte subsets identified so far^31^, classical CD14^+^CD16^−^ monocytes predominantly induced pro-IL-1β synthesis compared with intermediate CD14^+^CD16^+^ and nonclassical CD14^dim^CD16^+^ monocyte subsets (Fig. S3). In addition to pro-IL-1β induction, NF-κB-dependent priming step is also necessary for upregulation of NLRP3^32^. As seen in Fig. 1a, NLRP3 protein was present in monocyte lysates of unstimulated PBS control and its expression level was comparable between MSU crystal stimulation and PBS control until 1 hr after stimulation. These data suggest that MSU crystals, which have previously been exclusively known as an inflammasome inducer, might play a role as a priming stimulus for pro-IL-1β production.

Of note, calcium pyrophosphate dehydrate (CPPD), another type of endogenous crystal involved in the pathogenesis of pseudogout, also markedly increased pro-IL-1β protein levels in the cell lysates and the secretion of active IL-1β at 30 min post-stimulation (Fig. S4), suggesting that this priming ability is not limited to MSU crystals.

The rapid production of intracellular pro-IL-1β protein after stimulation with MSU crystals prompted us to test the possibility that pro-IL-1β synthesis might be post-transcriptionally controlled in monocytes. To explore this possibility, freshly-isolated human monocytes were stimulated with MSU crystals for 1 h in the absence or presence of the transcriptional inhibitor, actinomycin D (Act D) or the translational inhibitor, cycloheximide (CHX) (Fig. 1c). Pro-IL-1β production in human monocytes was markedly inhibited by co-treatment with CHX in the cells stimulated with MSU crystals but not in the PBS-treated control group (Fig. 1c; Lane 6 and 5, respectively), whereas Act D had no effect on pro-IL-1β production in response to MSU crystals (Fig. 1c; Lane 3 and 4, respectively). Furthermore, both MSU crystal-induced intracellular pro-IL-1β levels and extracellular release of IL-1β were remarkably suppressed at 1.25 μg/ml of CHX, and this suppression was gradationally attenuated by CHX in an inverse dose-dependent manner (Fig. 1d,e). These findings were corroborated with a real time RT-PCR assay showing that MSU crystals did not significantly affect pro-IL-1β mRNA levels in freshly-purified monocytes (Fig. 1f). Unlike monocytes, no production of pro-IL-1β was observed in mouse bone marrow-derived macrophages or in human monocyte-derived macrophages even 2-4 hr after stimulation with MSU (Fig. 1g and Fig. S5), suggesting that pro-IL-1β synthesis in monocytes and macrophages may be differentially controlled.

These results demonstrate that the induction of pro-IL-1β by MSU crystals is post-transcriptionally regulated in human monocytes implicating a possible role of MSU crystals as a priming stimulus for pro-IL-1β production.

### MSU crystals stimulate pro-IL-1β and global protein synthesis via translational regulation in human monocytes

**Figure 2:**
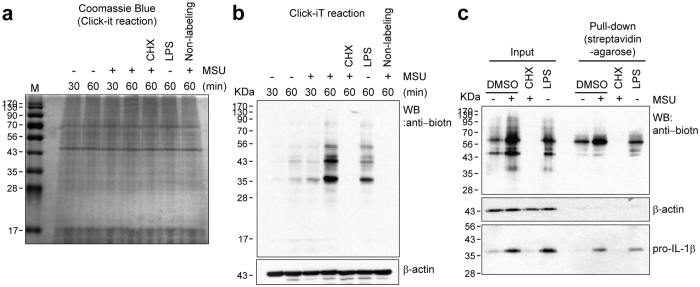
MSU crystals stimulate pro-IL-1β as well as global protein synthesis via translational regulation in human monocytes. (**a**) Human monocytes were pre-incubated for 90 min in methionine-free RPMI medium and metabolically labeled for another 60 min with the azide-containing methionine analog AHA (L-azidohomoalaine), followed by treatment with PBS (as vehicle control), MSU crystals (400 μg/ml), or LPS (100 ng/ml) in the absence or the presence of CHX for the indicated times. After incubation, cells lysates were prepared and the newly synthesized, AHA-incorporated proteins were cross-linked to alkyne-derivatized biotin by a copper-catalyzed cycloaddition (Click-iT reaction). The non-labeling sample did not have alkyne-derivatized biotin added (negative control). Biotin-conjugated newly-synthesized proteins were collected using streptavidin-agarose for immunoblot analysis of intracellular pro-IL-1β.After polyacrylamide gel electrophoresis, Coomassie blue staining was used to analyze the amount of total protein in each Click-iT-reacted sample. (**b**) Newly-synthesized proteins containing biotin-conjugated AHA were detected with streptavidin-HRP. (**c**) Biotin-conjugated newly-synthesized proteins (presented as “Input”) were collected using streptavidin-agarose (presented as “Pull-down”) and the indicated proteins were examined by immunoblot analysis using streptavidin-HRP (upper panel) or anti-IL-1β antibody (lower panel). β-actin was used as an internal control (**c**,**d**). (**a**–**c**) Data are representative of two independent experiments with two different donors.

To further investigate the underlying mechanism involved in the post-transcriptional regulation of pro-IL-1β in monocytes, we utilized metabolic labeling and pull-down assays by which newly synthesized pro-IL-1β protein could be directly detected and evaluated after MSU crystal stimulation. Coomassie blue staining clearly showed that the amount of total protein was comparable among different treatment groups (Fig. 2a). However, stimulation with MSU crystals led to rapid incorporation of the methionine analog indicating enhanced global protein synthesis, whereas protein synthesis in PBS-treated monocytes was at a basal level. As expected, CHX treatment wholly inhibited protein synthesis (lane 5, Fig. 2b). Consistent with a previous report in which new protein synthesis was rapidly and massively enhanced in murine dendritic cells in response to lipopolysaccharides (LPS)^33^, LPS-stimulated monocytes induced new protein synthesis at a similar rate to MSU crystal-stimulated monocytes (Lane 6 and 4, respectively, Fig. 2b).

To more specifically evaluate the level of newly synthesized pro-IL-1β proteins among globally synthesized proteins, biotin-conjugated proteins were collected by pull-down using streptavidin-agarose and the amount of pro-IL-1β was evaluated by immunoblotting (Fig. 2c). As shown in Fig. 2c, MSU crystals robustly triggered pro-IL-1β protein synthesis compared to the LPS-stimulated control, whereas synthesis of pro-IL-1β protein was completely repressed by CHX. Newly synthesized pro-IL-1β proteins were obviously observed as low as 100 μg/ml MSU crystal treatment and this pro-IL-1β synthesis was enhanced by MSU crystals in a dose-dependent manner (Fig. S6). These data strongly support the finding that MSU crystals stimulate pro-IL-1β production at the level of translation in human monocytes.

### MSU crystals stimulate signaling pathways involved in translational initiation in innate cells

**Figure 3:**
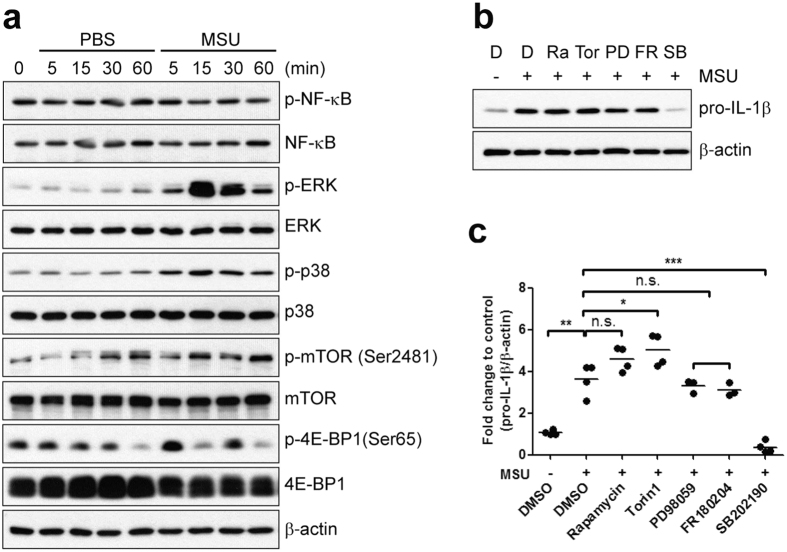
MSU crystal-induced production of pro-IL-1β is controlled by p38 MAP kinase signaling pathways. **(a**) Human primary monocytes were stimulated with PBS or MSU crystals (400 μg/ml) for the indicated times. Phosphorylation of NF-κB, ERK, p38, mTOR, and 4E-BP1 was assessed in monocyte lysates by immunoblot analysis with phospho-specific and total protein-specific antibodies. (**b**) Monocytes were stimulated with MSU crystals in the presence of inhibitors: 50 nM rapamycin (Ra: allosteric mTOR inhibitor), 50 nM Torin1 (Tor: selective mTOR C1/2 inhibitor), 20 μM PD98059 (PD: a potent inhibitor of MEK1), 10 μM FR180204 (FR: selective ERK1/2 inhibitor), 5 μM SB202190 (p38α and β isoform selective inhibitor), and DMSO (D: vehicle control). Monocytes were pretreated with these inhibitors for 30 min prior to stimulation with MSU crystals for 60 min. Cell lysates were analyzed by immunoblot for the effect of the inhibitors on pro-IL-1β synthesis. Intensities between upper and lower blots were normalized to vehicle control (1^st^ lane). (**c**) The effect of the inhibitors on pro-IL-1β synthesis is presented as fold change compared with vehicle control (1^st^ lane). Values were normalized to β-actin. The scatter plots show the mean from three to four independent experiments with three to four different donors. N.S. indicates not significant, *p < 0.05 and ***p < 0.005 by unpaired *t*-test in (**c**).

Translation initiation is the rate-limiting step in protein synthesis, and one of the molecules that acts as a key controller is mammalian target of rapamycin complex 1 (mTORC1)^34^. The best-described downstream target of mTORC1 linked to translational control is the 4E-BP1, which inhibits the translation-initiation factor, eIF4E, by sequestration^35^. eIF4E is the cap-binding subunit of the eIF4F complex and its activity is also modulated at the phosphorylation level by MAPK-interacting protein kinases 1 (Mnk1), which is in turn regulated via both MEK/ERK and p38^36^,^37^,^38^. To explore the molecular mechanism by which MSU crystals stimulate pro-IL-1β synthesis, we next investigated the effects of MSU crystals on various signaling pathways known to be involved with translation initiation in innate cells. As seen in Fig. 3a, MSU crystals rapidly activated ERK1/2 and p38 MAPKs and also induced phosphorylation of mTOR and 4E-BP1, whereas NF-кB activity was not changed at 1 hour post-stimulation with MSU crystals.

To address which signaling pathway(s) is responsible for regulating pro-IL-1β synthesis in MSU crystal-stimulated human monocytes, inhibitors were used to pretreat the cells before stimulation with MSU crystals. Although both ERK1/2 and p38 MAPKs were rapidly phosphorylated following stimulation with MSU crystals (Fig. 3a), pro-IL-1β production in MSU crystal-stimulated human monocytes was dramatically suppressed by treatment with the p38 MAPK inhibitor, SB202190, but not by ERK1/2 inhibitors, PD98059 and FR180204 (Fig. 3b,c). Moreover, MSU crystal-stimulated pro-IL-1β production was not affected by the presence of rapamycin, while Torin 1, a selective mTOR inhibitor, slightly but significantly increased pro-IL-1β production (1.4-fold) compared with vehicle control (Fig. 3b,c). These results demonstrate that MSU crystals activate both mTOR and MAPK signaling pathways for translation initiation of global proteins, and further, that p38 MAPK signaling is critical for MSU crystal-induced pro-IL-1β synthesis in human monocytes. Of note, the inhibitory effect of PD98059 and SB202190 on pro-IL-1β production was entirely opposite in MSU crystal-stimulated THP-1 cells compared with that in primary human monocytes (Fig. 3b and S7). We found that PD98059 inhibited MSU crystal-induced production of pro-IL-1β in a dose-dependent manner in THP-1 cells and completely blocked secretion of mature IL-1β in culture supernatants, whereas SB202190 did not affect intracellular pro-IL-1β production and only moderately repressed secretion of mature IL-1β (Fig. S7). This suggests that signal pathways upstream of IL-1β production differ between human primary monocytes and PMA-treated THP-1 cells.

### MSU crystal-stimulated pro-IL-1β protein synthesis is dependent on the p38 MAPK signaling pathway, but not the mTOR signaling pathway

**Figure 4:**
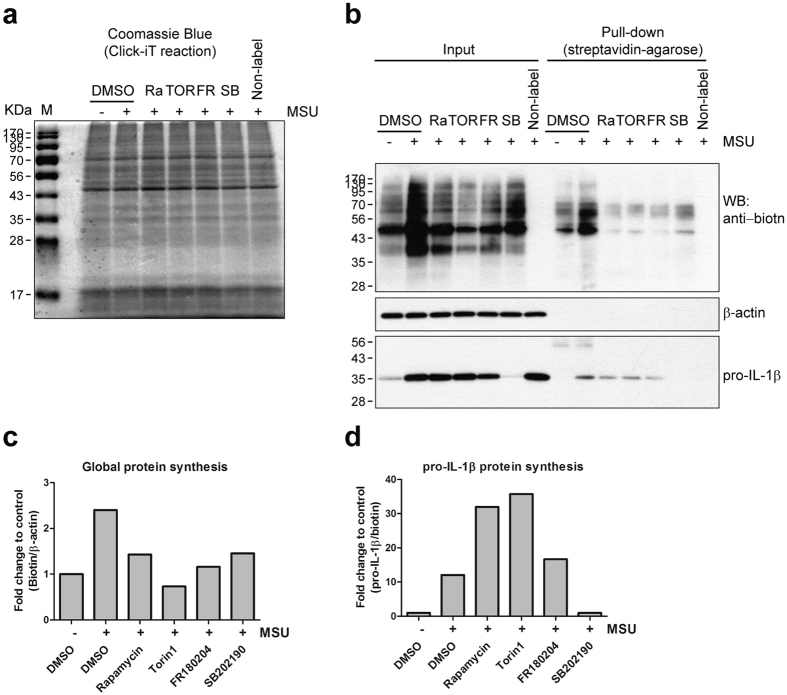
MSU crystal-stimulated pro-IL-1β protein synthesis is dependent on p38 MAPK, but not on mTOR. Click-iT^®^ Labeling and pull-down assay of biotinylated proteins was performed as previously described in Fig. 2. Human monocytes were stimulated with MSU crystals for 60 min after 30 min pretreatment with inhibitors: Rapamycin (50 nM), Torin 1 (50 nM), PD98059 (20 μM), FR180204 (10 μM), SB202190 (5 μM), and DMSO (vehicle control). (**a**) Coomassie blue staining was used to analyze the amount of total proteins in each Click-iT-reacted sample after polyacrylamide gel electrophoresis. (**b**) Biotin-conjugated newly-synthesized proteins (Input) were collected by streptavidin-agarose (Pull-down) and the indicated proteins were examined by immunoblot analysis using streptavidin-HRP (upper panel) or anti-IL-1β antibody (lower panel). (**c**,**d**) The effect of the inhibitors on the synthesis of nascent global proteins (**c**) and pro-IL-1β protein (**d**) is presented as fold change compared with vehicle control (1^st^ lane). Global protein and pro-IL-1β protein were normalized to β-actin (Input) and total biotinylated protein (Pull-down), respectively. Data is representative of two independent experiments with two different donors.

Since MSU crystal-stimulated pro-IL-1β production was remarkably suppressed by SB202190 (Fig. 3b), we next examined whether pro-IL-1β synthesis in MSU crystal-stimulated monocytes is regulated downstream of p38 MAPK. After electrophoresis of biotin-conjugated protein samples, total protein was visualized by Coomassie blue staining and the levels were found to be similar among different inhibitor treatment groups (Fig. 4a). Further, immunoblotting analysis clearly shows that global protein synthesis and pro-IL-1β protein synthesis are differentially controlled by the mTOR and MAPK pathways (Fig. 4b–d). As shown in Fig. 4c,d, global protein synthesis, which was normalized to β-actin, was suppressed by all inhibitors used due to their role in translation regulation, whereas pro-IL-1β synthesis, which was normalized to total biotinylated proteins, was significantly repressed by SB202190 but not by mTOR or ERK inhibitors (Fig. 4b–d). CPPD crystal-stimulated pro-IL-1β protein synthesis was also selectively dependent on the p38 MAPK signaling pathway (Fig. S8A,B), suggesting that this p38 MAPK-mediated translation regulation is not limited to MSU crystals. Together, these findings show that the post-translational regulation of pro-IL-1β synthesis by MSU crystals is predominantly regulated via the p38 MAPK signaling pathway in primary human monocytes.

### MSU crystal-stimulated pro-IL-1β protein synthesis is regulated by translation initiation via the Mnk1/eIF4E pathway

**Figure 5:**
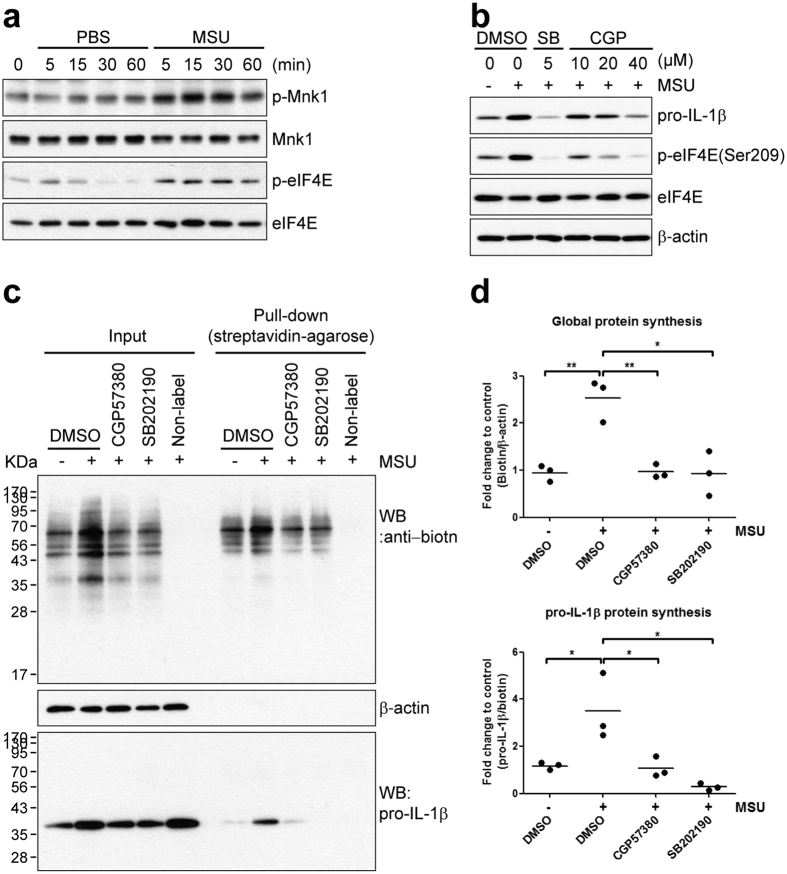
Inhibition of Mnk1, a p38 MAPK substrate, suppresses MSU crystal-stimulated pro-IL-1β and global protein synthesis. (**a**) Human primary monocytes were stimulated with PBS or MSU crystals (400 μg/ml) for the indicated times. Phosphorylation of MnK1 and eIF4E were assessed in monocyte lysates by immunoblot analysis with phospho-specific and total protein-specific antibodies. Data is representative of two independent experiments with two different donors. (**b**) Human monocytes were pre-treated for 30 min with the indicated concentrations of CGP57380 (Mnk1 inhibitor), SB202190 (5 μM), or DMSO (vehicle) prior to stimulation with MSU crystals (400 μg/ml) for 60 min. Cell lysates were analyzed by immunoblot for the effect of the inhibitors on pro-IL-1β synthesis. The values between upper and lower blots were normalized to vehicle control (1^st^ lane). Data is representative of two independent experiments with two different donors. (**c**) Biotin-conjugated newly-synthesized proteins (Input) were collected using streptavidin-agarose (Pull-down) and the indicated proteins were examined by immunoblot analysis using streptavidin-HRP (upper panel) or anti-IL-1β antibody (lower panel). Data is representative of three independent experiments with three different donors. (**d**) The inhibitory effect of the CGP57380 on the synthesis of nascent global proteins and pro-IL-1β protein is presented as fold change compared with vehicle control (1^st^ lane). The values of global and pro-IL-1β proteins were normalized to β-actin (Input) and total biotinylated protein (Pull-down), respectively. The scatter plots show the mean of three independent experiments with three different donors. N.S. indicates not significant, *p < 0.05 and **p < 0.01 by unpaired *t*-test in (**d**).

MSU-crystal induced pro-IL-1β synthesis is selectively and wholly suppressed at the post-transcriptional level by inhibition of p38 MAPK (Fig. 4c,d). Thus, we next analyzed pathways downstream of p38 MAPK that may contribute to MSU crystal-mediated pro-IL-1β synthesis in human monocytes. As seen in Fig. 5a, stimulation with MSU crystals results in the rapid activation of Mnk1, a direct target of p38 MAPK responsible for eIF4E phosphorylation. Since Mnk1-mediated eIF4E phosphorylation leads to enhanced translation of TNF-α mRNA in innate cells^39^,^40^, we further investigated the role of Mnk1 in pro-IL-1β synthesis using CGP57380, a selective inhibitor of Mnk1^41^. The immunoblotting data in Fig. 5b clearly shows that phosphorylation of eIF4E in response to stimulation with MSU crystals was gradually attenuated by CGP57380 in a dose-dependent manner, with an almost complete blockade at 40 μM (Fig. 5b middle panel). This repression of eIF4E phosphorylation led to the inhibition of intracellular pro-IL-1β synthesis in MSU crystal-stimulated human monocytes (Fig. 5b, uppermost panel). Indeed, inhibition of Mnk1 significantly suppressed both global protein and pro-IL-1β synthesis in response to MSU crystals (Fig. 5c,d), suggesting an essential role of enhanced translation initiation for MSU crystal-mediated pro-IL-1β synthesis. These results show that Mnk1 plays a key role in protein translation downstream of p38 MAPK activation in response to MSU crystal stimulation of human monocytes.

### MSU crystals increase pro-IL-1β mRNA stability via the p38 MAPK/MK2 signaling pathway

**Figure 6:**
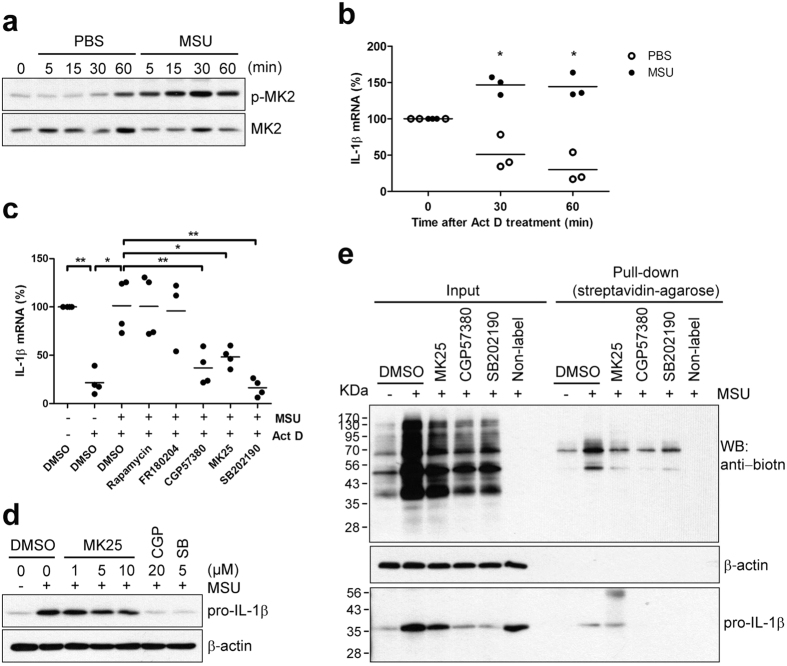
MSU crystals increase pro-IL-1β mRNA stability, which is controlled by p38 MAPK signaling pathway via MK2. (**a**) Human primary monocytes were stimulated with PBS or MSU crystals (400 μg/ml) for the indicated times. Phosphorylation of MK2 was assessed in monocyte lysates by immunoblot analysis with phospho-specific and total protein-specific antibodies. (**b**) Cells were treated with Act D (2.5 μg/ml) prior to stimulation with MSU crystals (400 μg/ml) or PBS for the indicated times. RNA was isolated from the monocytes and the level of pro-IL-1β transcription was determined using SYBR green qPCR. The level of the pro-IL-1β mRNA before treatment with Act D and MSU crystals was considered as 100%. Human GAPDH was used for normalization. The scatter plots show the mean in triplicate from three ndependent experiments with three different donors. (**c**) The effect of inhibitors including Rapamycin (50 nM), FR180204 (10 μM), CGP57380 (Mnk1 inhibitor, 20 μM), MK25 (MK2 inhibitor, 10 μM), and SB202190 (5 μM) on pro-IL-1β mRNA stability is presented as the % change compared with vehicle control (1^st^ lane). The scatter plots show the mean in triplicate from three to four independent experiments with three to four different donors. (**d**) Human monocytes were pre-treated for 30 min with the indicated concentrations of MK25 (MK2 inhibitor), CGP57380 (Mnk1 inhibitor), SB202190, or DMSO (vehicle) prior to stimulation with MSU crystals (400 μg/ml) for 60 min. Cell lysates were analyzed by immunoblot for pro-IL-1β production. The graph shows the mean ± SEM in triplicate from three independent experiments with three different donors. (**e**) Biotin-conjugated newly-synthesized proteins (Input) were collected using streptavidin-agarose (Pull-down) and the indicated proteins were examined by immunoblot analysis using streptavidin-HRP (upper panel) or anti-IL-1β antibody (lower panel). Blots are two independent experiments with two different donors. *p < 0.05 and **p < 0.01 by paired (**b**) or unpaired *t*-test (**c**).

Control of mRNA stability is another critical mechanism of post-transcriptional regulation that occurs downstream of the p38/MK2 signaling pathway^42^,^43^. As seen in Fig. 6a, MK2, a direct target of p38 MAPK, was rapidly activated in response to stimulation of cells with MSU crystals. Therefore, we investigated whether inhibition of p38 MAPK could also modulate pro-IL-1β mRNA stability. To this end, Act D was used as a general RNA synthesis inhibitor and the pro-IL-1β mRNA level was monitored at the indicated times in MSU crystal-stimulated monocytes. In PBS-treated control cells pro-IL-1β mRNA decays rapidly, with a half-life of approximately 30 min. Of note, stimulation with MSU crystals significantly stabilized pro-IL-1β mRNA, increasing the half-life 2.88 fold (Fig. 6b). This supports the finding that the total pro-IL-1β mRNA level was slightly increased in MSU crystal-stimulated monocytes compared with the PBS-treated control (Fig. 1f), and that mRNA turnover is rapid and robust in human monocytes. To address the underlying signaling pathway(s) controlling MSU crystal-induced stabilization of pro-IL-1β mRNA, cells were pretreated with specific pathway inhibitors before stimulation with MSU crystals in the presence of Act D. We found that MSU crystal-induced stabilization of pro-IL-1β mRNA was significantly suppressed by treatment with p38 MAPK inhibitor SB202190, Mnk1 inhibitor CGP57380, and MK2 inhibitor MK25, but not mTOR inhibitor rapamycin or the ERK 1/2 inhibitor FR180204 (Fig. 6c). We next examined whether the p38/MK2 pathway regulates MSU crystal-induced pro-IL-1β protein synthesis. Although pro-IL-1β mRNA was stabilized by MSU crystals through the p38/MK2 signaling pathway, the level of pro-IL-1β synthesis was only slightly affected by inhibition of MK2 activity compared with Mnk1 inhibitor or p38 MAPK inhibitor treatment (Fig. 6d). Indeed, inhibition of MK2 slightly decreased global protein synthesis but did not affect pro-IL-1β synthesis, whereas inhibition of Mnk1 significantly suppressed both global protein and pro-IL-1β synthesis in response to MSU crystals (Fig. 6e). This result demonstrates that the MSU crystal-activated p38 MAPK signaling pathway plays an important role in pro-IL-1β mRNA stability as well as in the translation initiation of pro-IL-1β mRNA.

## Discussion

The innate immune system is equipped with a variety of germline-encoded PPRs, which were originally identified as receptors recognizing microorganism-derived components known as pathogen-associated molecular patterns (PAMPs). Recently PPRs have also been shown to sense host cell-derived components (DAMPs)^1^,^4^. Sterile inflammation can be incited by a variety DAMPs, which are normally sequestered intracellularly, but are released into the extracellular environment by dying cells and damaged tissue in the absence of microbial infections^15^,^44^,^45^. Non-communicable chronic diseases that are potentiated by sterile inflammation are a major threat to human health^46^,^47^. Among numerous DAMPs, endogenous sterile crystals are directly responsible for the pathogenesis of several diseases including gout, pseudogout, Alzheimer’s disease and atherosclerosis. Therefore, understanding the cellular and molecular mechanisms underlying crystal-mediated sterile inflammation has become a priority^6^,^14^,^19^,^48^.

In agreement with previous reports^19^, our data clearly show MSU crystal-mediated release of active IL-1β (17 kD) into the supernatant of human primary monocytes (Fig. S1). However, the time point at which active IL-1β was initially observed in the supernatant was much earlier than expected based on previous reports using PMA-stimulated THP-1 cells^19^. More importantly, the intracellular pro-IL-1β protein level was markedly augmented as early as 60 min post-stimulation with MSU crystals (Fig. S1). While MSU crystals are well known as an important activator of the inflammasome (signal 2), recent work has demonstrated that sterile crystals can also induce pro-IL-1β during the priming step (signal 1)^49^,^50^,^51^; however, the mechanism underlying the generation of this signal has not been clear.

One significant finding in the present study is the rapid production of pro-IL-1β protein in response to stimulation of primary human monocytes with MSU crystals, with the notable absence of an effect on its mRNA level. This suggests the involvement of post-transcriptional controls (Fig. 1). Further, activation of NF-κB, which leads to transcriptional induction of pro-IL-1β mRNA synthesis during the priming step, was not observed in monocytes upon MSU stimulation (Fig. 3a). Therefore, as a consequence, MSU crystals did not significantly affect pro-IL-1β mRNA levels in freshly-purified monocytes (Fig. 1f). In spite of obvious early induction of pro-IL-1β in response to MSU crystals, our data also showed a time-dependent upregulation of pro-IL-1β in PBS control group. Human monocytes are highly sensitive to many environmental stimuli such as TLR agonists, DAMP, even attachment. Furthermore, their mechanism of IL-1β secretion is very unique compared with human macrophage and murine monocyte^52^. It was reported that adhesion of human monocytes to culture dish as well as extracellular matrix component such as fibronectin and collagen, induced the synthesis of large amounts of IL-1β mRNA^53^,^54^. Adhesion of human monocytes stimulates cytoskeletal rearrangement which increases IL-1β mRNA stability and induces a redistribution of IL-1β transcripts. Substantial evidence supports that cytoskeleton participates in spatially and temporally organizing key components of the translational apparatus and regulation of translation^55^.

We optimized the experimental protocol where monocytes were handled and kept in polypropylene tube to minimize attachment to its surface. Of course, we could not completely inhibit attachment of monocytes to the bottom of tube during the incubation but markedly reduced (data not shown). Presumably, the time-dependent upregulation of pro-IL-1β, especially after 30-60 min in PBS control group might result from attachment-mediated monocyte activation. However, it should be noted that MSU stimulation significantly upregulates pro-IL-1β protein synthesis compared with PBS control (Fig. 1a,b).

In contrast to our finding, several studies reported lack of effect of MSU crystals in primary PBMC on IL-1β production in the absence of costimulatory ligands as priming signal^56^. One possible explanation on this discrepancy is that we investigated on early priming of pro-IL-1β in purified primary monocytes not in PBMC. Only around 5% of PBMC is monocyte subset in humans. Since we used purified monocytes for all experiments, the amount of IL-1β in our experiments could be much higher than that produced by MSU-stimulated PBMC. Indeed, van der Meer and colleagues also showed that around 100 pg/ml of IL-1β was secreted from PBMC in response to 100 - 1,000 μg/ml of MSU^57^. Furthermore, Tschopp and colleagues showed that MSU crystals alone could induce IL-1β secretion by purified human monocytes^19^. As above-mentioned, adhesion of human monocytes stimulates cytoskeletal rearrangement which increases IL-1β mRNA stability and induces a redistribution of IL-1β transcripts^55^. Monocyte purification steps are likely to give more changes to make them adhere to plastic ware. Indeed, adherence of monocytes to endothelium is an early step in the development of arteriosclerotic lesions and infection and has been suggested to “prime” monocytes for their role in inflammatory processes. In very recent, two papers suggested one-step non-canonical (or alternative) inflammasome activation in human monocytes^58^,^59^.

CPPD crystals, another type of endogenous pathogenic particulate, also greatly enhanced pro-IL-1β protein levels in cell lysates as early as 30 min post-stimulation (Fig. S4). Therefore, this suggests that post-transcriptional control might play an important role during pro-IL-1β synthesis in crystal-induced inflammation in humans.

Considering that the IL-1β level in the culture supernatant is dependent on intracellular pro-IL-1β levels following stimulation with MSU crystals (Fig. 1d,e), this post-transcriptional control could be a rate-limiting step in the production of IL-1β. Our pull-down assay combined with metabolic labeling of newly-synthesized proteins directly showed that MSU crystals stimulated rapid and robust global protein synthesis as well as pro-IL-1β synthesis (Fig. 2). Our initial finding that MSU post-transcriptionally induces global protein synthesis was corroborated by immunoblot analysis of translational control-associated major signaling pathways, including mTOR, p38, and ERK molecules. Further, our immunoblot data clearly demonstrate that MSU stimulation rapidly induces the phosphorylation of these signaling molecules, but not of NF-kB, which is a major transcriptional trigger for pro-IL-1β mRNA synthesis (Fig. 3a). More importantly, specific inhibitors of each signaling pathway dramatically repressed synthesis of global proteins following MSU stimulation (Fig. 4b,c). As seen in Fig. 3a, activity of mTOR and 4E-BP1 exhibited a bimodal upregulation of expression. Although mTOR signaling is a major pathway for 4E-BP1 phosphorylation, other signaling pathways such as the PI3K and ERK pathway also lead to 4E-BP1 phosphorylation^60^,^61^,^62^,^63^. It is possible that MSU-mediated phosphorylation of ERK at early time points may affect the 4E-BP1 phosphorylation and cause the bimodal phosphorylation of 4E-BP1 in MSU-stimulated monocytes (Fig. 3a).

Of interest, MSU crystal-stimulated pro-IL-1β synthesis is selectively repressed by both the p38 MAPK inhibitor and the Mnk1 inhibitor, but not by the inhibition of ERK or mTOR. These results suggested that MSU crystal-induced global protein synthesis and pro-IL-1β synthesis are regulated by separated signaling pathways. However, phosphorylation of eIF4E is crucial for the initiation of translation in both pathways (Figs 4b–d and 5c,d). Unexpectedly, pre-treatment of Torin 1 slightly but significantly increased MSU-mediated pro-IL-1β synthesis in human monocytes, suggesting that mTOR might negatively regulate of pro-IL-1β synthesis (Fig. 3c). It has been reported that mTORC2 can bind DUSP10, the p38 MAPK phosphatase, and phosphorylates DUSP10 on serine residues 224 and 230. These phosphorylation events block DUSP10 turnover by increasing its stabilization and result in inhibition of p38 activity^64^. Supposedly, inhibition of mTORC2 by Torin 1 downregulates DUSP10 stability and consequently, sustains or enhances p38 MAPK activity. It may leads to increase of p38-mediated post-transcriptional pro-IL-1β synthesis in human monocytes.

In agreement with the previous finding^65^, inhibition of the ERK pathway by the selective MEK1 inhibitor PD98059 completely blocks pro-IL-1β production and secretion of IL-1β in MSU crystal-stimulated THP-1 cells (Fig. S7), whereas MSU crystal-induced pro-IL-1β production is prevented only by inhibition of p38 MAPK in human primary monocytes. These results provide evidence that MSU crystals induce pro-IL-1β and IL-1β production in both human monocytes and THP-1 cells, however they are differentially controlled by unique signaling pathways^22^,^23^. Further, MSU crystals induced pro-IL-1β in primary human and murine monocytes, but not in differentiated macrophages (Figs 1g and S5).

Phagocytosis of large crystals, such as MSU, causes lysosomal damage, resulting in leakage of a variety of lysosomal contents into the cytosol^14^,^66^,^67^,^68^. These contents directly and indirectly contribute to NALP3 inflammasome activation. In addition to phagocytosis, several mechanisms has been suggested for how MSU crystals are recognized by surface molecules on immune cells and initiate particular signaling pathways, which, given the present study, are presumably associated with pro-IL-1β expression. One study reported that CD14 could directly bind to MSU. Further, CD14^−/−^ macrophages showed attenuated phosphorylation of p38 and IL-1β release in response to MSU, and decreased pro-IL-1β protein expression^69^. More recently, Shi and colleagues demonstrated that MSU crystals can directly engage cholesterol components in cellular membranes of bone-marrow derived dendritic cells and subsequently cause lipid sorting. This event leads to the recruitment of Syk to membrane-associated ITAMs, which in turn phosphorylates PI3K upstream of mTOR^70^. It is important to note that these mechanisms might be important for post-transcriptional control of both global protein and pro-IL-1β (Fig. 1).

The mRNAs of many inflammatory cytokines including TNF-α, IL-1β and IL-10 are potentially unstable^71^. Thus, the regulation of mRNA stability is an important post-transcriptional mechanism of cytokine production^25^. Consistent with previous findings, pro-IL-1β mRNA is relatively rapidly decayed at the resting state, showing a half-life of around 30 min. Our data revealed that MSU crystal stimulation increased the mRNA stability of IL-1β (Fig. 6b) through increased MK2 phosphorylation. Of note, activation of Mnk1 or MK2, substrates of p38, is associated with increased mRNA stability, but only Mnk1 has been selectively linked to enhanced translational initiation of IL-1β (Fig. 6d). Thus, translational control appears to play a predominant role in MSU crystal-induced pro-IL-1β synthesis in primary human monocytes.

Although our results show that the p38/Mnk1/eIF4E axis plays a critical role in MSU crystal-induced pro-IL-1β synthesis, inhibition of the p38 pathway also blocked global protein synthesis. The post-transcriptional control of cytokine production requires an integrated mechanism of both general and cytokine-encoding mRNA-specific aspects of mRNA metabolism. Translation of most inflammatory cytokine mRNAs is regulated in a transcript-specific manner through interactions between specific RNA-binding proteins and adenylate/uridylate-rich elements (AREs) located in the 3′untranslated region (3′-UTR)^71^. Further, the p38 pathway stabilizes the mRNAs of inflammatory response proteins^72^ and promotes their translation^73^ through AREs^71^. Several different ARE-binding proteins (ARE-BPs) are recruited to AREs that can positively or negative regulate mRNA stability and/or translation. Two such ARE-BPs reportedly bind to the IL-1β mRNA. One is the mRNA destabilization factor tristeraprolin (TTP), which is phosphorylated following activation of p38 MAPK decreasing its RNA-binding activity^27^. The other is AUF1/hnRNP D, which is also an ARE-binding destabilizing factor. AUF1 knockout mice display symptoms of severe endotoxic shock and specific overproduction of TNF-α and IL-1β, which is the result of an inability to rapidly degrade these mRNAs in macrophages following LPS challenge^74^. It is possible that MSU crystal may affect the IL-1β mRNA binding activity of TTP or AUF and that p38 plays a role in this; however, identification of any detailed molecular linkage between the p38 pathway and ARE-mediated pro-IL-1β expression will require further investigation.

**Figure 7:**
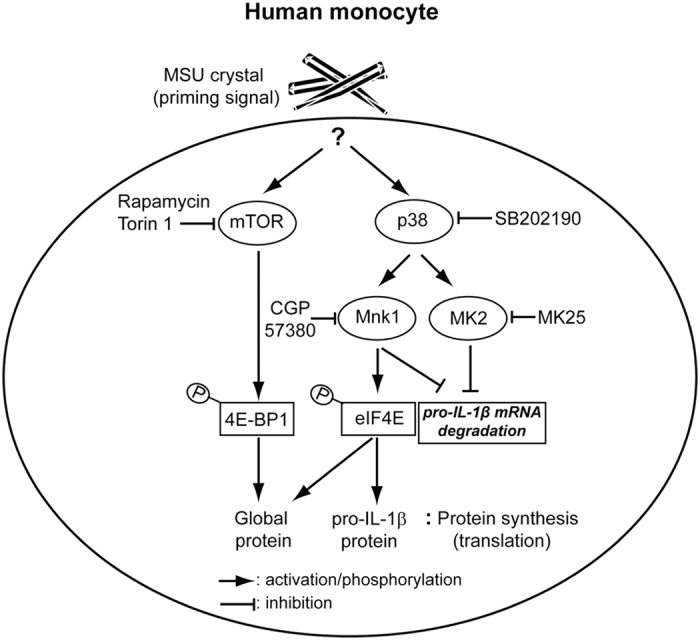
Proposed model of MSU crystal-mediated post-transcriptional regulation of pro-IL-1β synthesis in primary human monocytes. Upon MSU crystal stimulation, mTOR and p38 MAPK are rapidly phosphorylated in human primary monocytes, leading to the phosphorylation of 4E-BP1, Mnk1, MK2, and eIF4E, which are all predominant post-transcriptional regulators. Translational initiation and mRNA stability of pro-IL-1β in human monocytes is selectively regulated by p38 MAPK signaling pathway via MnK1 and MK2 phosphorylation, but not the mTOR signaling pathway.

Our studies demonstrate that endogenous sterile particulates such as MSU crystals induce the rapid synthesis of pro-IL-1β proteins in human primary monocytes through post-transcriptional mechanisms. Moreover, the major signaling pathways known to control post-transcriptional events are preferentially activated in MSU crystal-stimulated monocytes. Metabolic labeling and pull-down assays for nascent proteins clearly showed that MSU crystal-induced pro-IL-1β synthesis is selectively dependent on the p38 MAPK pathway, whereas global protein synthesis is mediated by the mTOR, ERK1/2, and p38 pathways. Inhibition of Mnk1, a substrate of p38, by CGP57380 markedly blocked MSU crystal-induced pro-IL-1β synthesis downstream of eIF4E phosphorylation. Furthermore, the p38 MAPK pathway was also critical for stabilization of pro-IL-1β mRNA through phosphorylation of MK2 and Mnk1 upon MSU stimulation. Thus, p38 MAPK-mediated post-transcriptional regulation plays a central role during the rapid synthesis of pro-IL-1β in response to MSU crystals, which is an essential step for active IL-1β production in human monocytes (Fig. 7). This new mechanism may help to explain the pathogenesis of gout and might result in the identification of new therapeutic targets for this and other crystal-induced sterile inflammatory diseases.

## Materials and Methods

### Antibodies and reagents

The following antibodies (Abs) and reagents were used in present studies: anti-IL-1β (human specific, mouse specific, and human & mouse specific), anti-phospho NF-κB, anti-NF-κB, anti-phospho ERK, anti-ERK, anti-phospho p38, anti-p38, anti-phospho 4E-BP1, anti-4E-BP1, anti-phospho Mnk1, anti-Mnk1, anti-phospho eIF4E, anti-eIF4E, anti-phospho MK2, anti-MK2, and anti-streptavidin-HRP antibodies were purchased from cell signaling technology (Danvers, MA). Anti-β-actin, Dimethyl sulfoxide, Actinomycin D, Cyclohexamide, ultrapure *E. coli* O111:B4 LPS, Rapamycin, PD98059, and SB202190 were purchased from Sigma-Aldrich (St. Louis, MO). Anti-NLRP3 antibody was purchased from Adipogen (San Diego, CA). Torin 1 and FR180204 were purchased from Merck Millipore (Billerica, MA). CGP57380 and MK25 were purchased from TOCRIS (Ellisville, MO, USA) and Cayman Chemical (Ann Arbor, MI), respectively. Recombinant human M-CSF and mouse M-CSF were purchased from R&D system (Minneapolis, MN).

### Preparation and stimulation of human monocytes and human monocyte-derived macrophages

The study protocols were reviewed and approved by the institutional review board of the institutional review board of Seoul National University Hospital (IRB No. C-1109-055-378 and C-1306-002-491). Peripheral blood of all healthy volunteers was drawn after obtaining the written informed consent at Seoul National College of Medicine. The methods were performed in accordance with the approved guidelines. Mononuclear cells were isolated from peripheral blood by density gradient centrifugation (Bicoll separating solution; BIOCHROM Inc, Cambridge, UK). Monocytes were positively separated from peripheral blood mononuclear cells (PBMC) with anti-CD14 magnetic beads (Miltenyi Biotec Inc, Auburn, CA) and were cultured in serum free RPMI 1640 medium supplemented with 100 units/ml penicillin and 100 μg/ml streptomycin (Gibco, Grand Island, NY). To minimize the effect of adhesion on pro-IL-1 β production, purified monocytes were incubated in the polypropylene round-bottom tube (Corning, NY, USA) and stimulated for the indicated time with 400 μg/ml monosodium urate (MSU) or 400 μg/ml calcium pyrophosphate dehydrate (CPPD) crystals (InvivoGen, San Diego, CA). Endotoxin levels in MSU crystal preparations, as assessed by *Limulus* amoebocyte cell lysate assay (Lonza, Walkersville, MD, USA), were less than 0.05 endotoxin EU/ml. Human monocyte-derived macrophages (MDMs) were obtained after differentiation from monocytes by incubation with 50 ng/ml rhM-CSF in RPMI medium supplemented with antibiotics, 2 mM L-glutamine (Gibco, Grand Island, NY), and 10% FBS (Biowest, France). After 6 d, the supernatant was removed, washed and cells were stimulated with MSU crystal (400 μg/ml) or ultrapure *E. coli* O111:B4 LPS (100 ng/ml) in serum free RPMI1640 medium.

### Mouse bone marrow-derived macrophage preparation and stimulation

Male C57BL/6 mice, aged 8 weeks, were obtained from the KOATECH Company Ltd (Gyeonggi-do, Korea) and were used for the generation of mouse bone marrow-derived macrophages (BMDMs). Mouse handling and the experimental procedures were approved by the Seoul National University Institutional Animal Care and Use Committees (permit numbers: SNU-150612-4) and were performed in accordance with the approved guidelines. The mouse whole bone marrow cells were isolated by flushing the marrow space of the femurs and tibiae and the non-adherent cells were cultured with M-CSF (25 ng/ml) in DMEM medium supplemented with antibiotics and 10% FBS. After 6 days, BMDMs were detached from 100 mm dish and counted, seeded in 12 well culture plate, and culture was continued for another 1 day. The supernatant was removed, washed and cells were stimulated with MSU crystal (400 μg/ml) or ultrapure *E. coli* O111:B4 LPS (100 ng/ml) in serum free DMEM medium.

### Detection of newly synthesized global proteins and pro-IL-1β

Newly synthesized proteins were detected by using the Click-iT method (Life Technologies, Grand Island, NY) according to the manufacturer’s instructions. Purified human monocytes were incubated in methionine-free RPMI 1640 (Gibco, Grand Island, NY) for 90 min before being metabolically labeled with the nonradioactive, azide-containing methionine analog AHA (L-azidohomoalaine), followed by stimulation with MSU crystal in the absence or presence of inhibitors for the indicated time. After incubation, the cells were pelleted by centrifugation and lysed in 50 mM Tris-HCl, pH 8.0, 1% SDS, and complete protease inhibitor cocktail (Pierce, Rockfod, IL). The newly synthesized, AHA-incorporated proteins were cross-linked to alkyne-derivatized biotin (Life Technologies, Grand Island, NY) by a copper-catalyzed cycloaddition using Click-iT protein reaction buffer kit (Life Technologies, Grand Island, NY). The labeled samples were precipitated using methanol/chloroform/water and the precipitated pellet was re-solubilized with 1% SDS solution plus complete protease inhibitor cocktail. The SDS was then quenched with 1 volume of NETFD buffer (100 mM NaCl, 50 mM Tris-HCl pH 7.4, 5 mM EDTA, 6% NP-40) plus protease inhibitors. Biotin-crosslinked proteins were mixed with streptavidin-agarose bead slurry (Pierce, Rockfod, IL) in spin column, and incubated at room temperature for 30 min. After spin down and washing, matrix-bound proteins were eluted into 2 × laemmil buffer by boiling. Total protein inputs and affinity-purified fractions were separated by SDS-PAGE and transfer of proteins onto the Polyvinylidene difluoride (PVDF) membrane, streptavidin-HRP was applied to the membranes to detect newly synthesized proteins and pro-IL-1β was detected by immunoblot analysis.

### Real-time RT-PCR and pro-IL-1β mRNA stability assay

Total RNA was isolated from human monocytes according to the manufacturer’s instructions using Qiagen RNeasy Plus Mini kit (Qiagen, Hilden, Germany). Quantitative real-time RT-PCR (qPCR) was performed in triplicate on a 7500 PCR system (Applied Biosystems, Grand Island, NY) and a SYBR green qPCR kit (Bioline, Taunton, MA) using the following primers; human GAPDH, forward 5′- GGAGCCAAAAGGGTCATCAT-3′ and reverse 5′- GTGATGGCATGGACTGTGGT-3′; and human IL-1β, forward 5′- CACGATGCACCTGTACGATCA-3′ and reverse 5′- GTTGCTCCATATCCTGTCCCT-3′. The level of gene expression was normalized to the expression of GAPDH. The comparative C_T_ method (*ΔΔ*C_T_) was used for the quantification of gene expression.

For the pro-IL-1β mRNA stability assay, freshly purified human monocytes were incubated with serum free RPMI 1640 in the presence of 2.5 μg/ml Act D (Sigma-Aldrich), followed by treatment with MSU crystals (400 μg/ml) for the indicated time. RNA was isolated from the monocytes and the level of pro-IL-1β transcription was determined with SYBR green qPCR as described above.

### Enzyme-linked immunosorbent assay (ELISA)

Cell culture supernatants were collected and centrifuged at 13,000 rpm for 5 min and the amount of IL-1β was quantified using commercial ELISA kit (eBioscience, San Diego, CA). The measurement of OD (Optical density) was performed using the Infinite 200 Pro Multimode microplate reader (Tecan, Seestrasse, Switzerland).

### Immunoblot analysis

For immunoblot analysis, human monocytes were lysed in lysis buffer (1% SDS, 100 mM, Tris pH8.0). Lysis buffer also contained protease and phosphatase inhibitor cocktail (Cell signaling, Canvers, MA). For the protein concentration from cultured medium, we used methanol and chloroform precipitation. Precipitated supernatants and total cell lysates were boiled with Laemmli sample buffer (×2 and ×5) and then run in a 10% or 12%SDS-PAGE. After electrophoresis, the proteins were transferred onto a PVDF membrane (Bio-rad, Hercules, CA), which was then blocked for 1 h with 5% BSA in Tris-buffered saline solution (TBS), containing 0.1% Tween 20. The membrane was incubated overnight with the respective primary antibodies at 4 °C, and then incubated with HRP-conjugated rabbit/mouse anti-IgG (Cell signaling, Canvers, MA) for 1 h at room temperature. Protein bands were detected by enhanced chemiluminescence (Millipore, Billerica, MA) on AGFA CP-BU x-ray films. Intensity of a band was quantified by ImageJ (National Institutes of Health, Bethesda, MD).

### Statistical analysis

Two-tailed paired *t*-test or unpaired t-test was done to analyze data using Prism 5 software (GraphPad Software Inc, La Jolla, CA) as indicated in the figure legends. *P* values of less than 0.05 were considered statistically significant. Results were obtained at least two or three separate experiments and expressed as mean ± SEM.

## Additional Information

**How to cite this article**: Chung, Y.-H. *et al*. Monosodium urate crystal-induced pro-interleukin-1β production is post-transcriptionally regulated via the p38 signaling pathway in human monocytes. *Sci. Rep.* **6**, 34533; doi: 10.1038/srep34533 (2016).

## Supplementary Material

Supplementary Information
