# Supplementary material for: Monosodium urate crystal-induced pro-interleukin-1β production is post-transcriptionally regulated via the p38 signaling pathway in human monocytes
Source: Sci Rep. 2016 Oct 3;6:34533. doi: 10.1038/srep34533 (PMC5046103; doi:10.1038/srep34533)
Supplement: Supplementary Information [file srep34533-s1.pdf]

Title: *Monosodium urate crystal-induced pro-interleukin-1β production is post-transcriptionally regulated via the p38 signaling pathway in human monocytes*

Authors: Yeon-Ho Chung, Dong-Hyun Kim and Won-Woo Lee

Supplementary figures

Supplementary figure 1

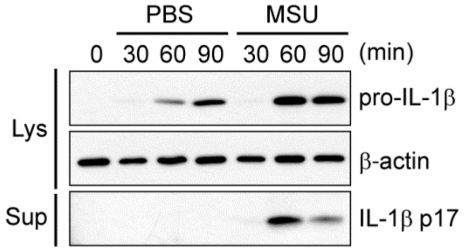

Figure S1. MSU crystals induce rapid production of pro-IL-1β and secretion of mature IL-1β proteins in primary human monocytes. Human monocytes were stimulated with MSU crystals (400 µg/ml) or PBS for the indicated time periods. Cell lysates (Lys) were analyzed for the presence of pro-IL-1β and supernatants (Sup) were analyzed for the presence of mature IL-1β (p17) by immunoblot analysis. Data is representative of two independent experiments.

Supplementary figure 2

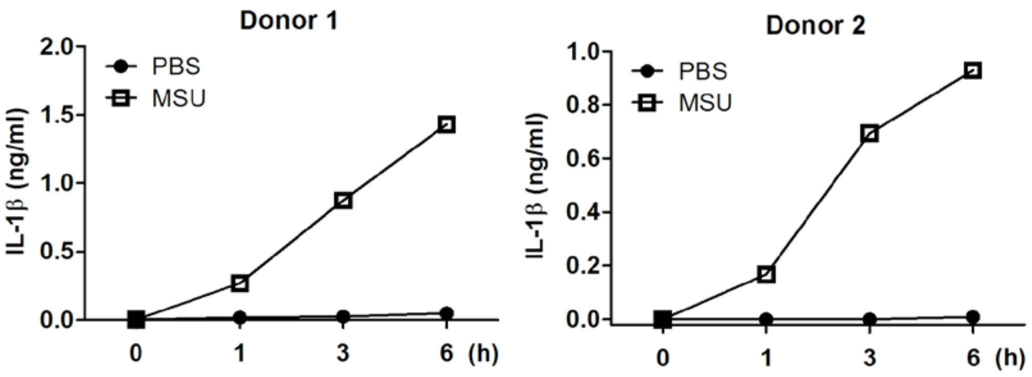

Figure S2. The early induction of pro-IL-1β synthesis by monocytes stimulated with MSU crystals leads to increased secretion of IL-1β at later time points. Human monocytes were stimulated with MSU crystals (400 µg/ml) or PBS for the indicated time periods. The amount of IL-1β in the culture supernatants was quantified by a conventional ELISA. Data are two independent experiments with two different donors.

Supplementary figure 3

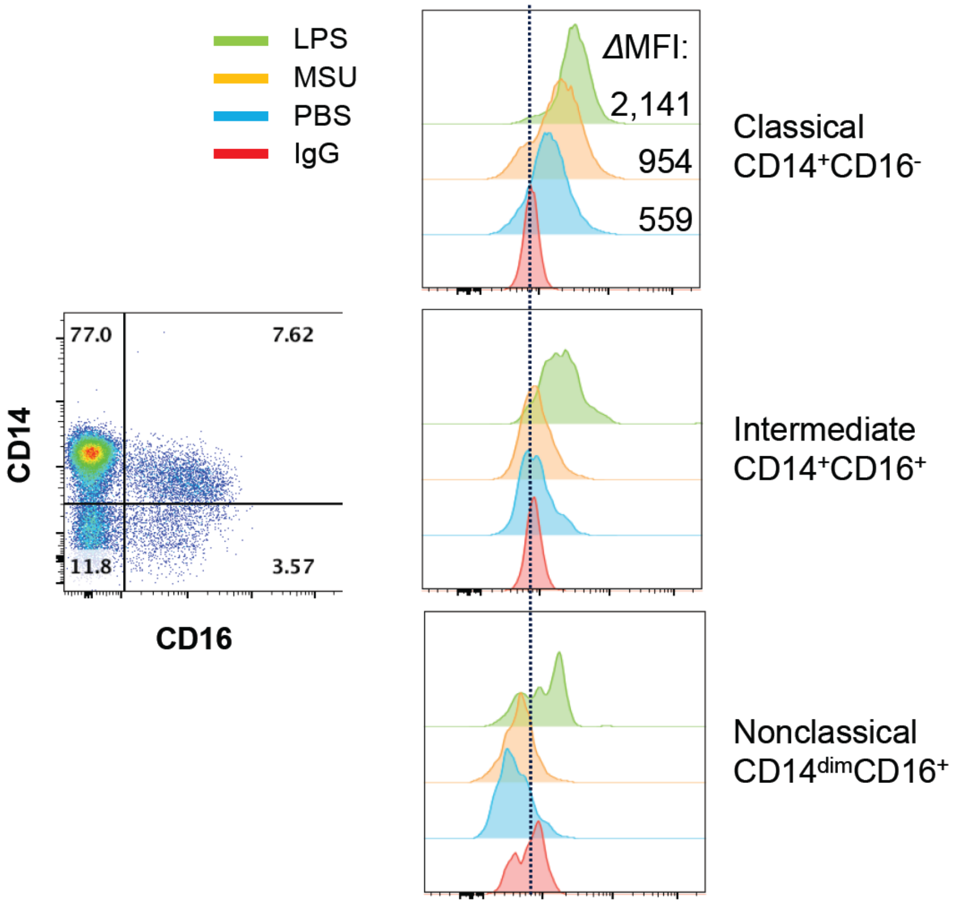

Figure S3. MSU-mediated early induction of pro-IL-1β synthesis predominantly occurs in classical CD14^{+}CD16^{-} monocytes. PBMC were stimulated for 1 hr with MSU (400 µg/ml), LPS (50 ng/ml) as positive control or PBS as vehicle control. For intracellular cytokine staining, stimulated PBMC were stained with anti-CD14 and anti-CD16 antibodies for 30 min, followed by fixation and permeabilization. The cells were stained with anti-pro-IL-1β (Cell signaling technology; Danvers, MA) and analyzed using a BD LSRFortessa. Data is representative of three independent experiments with three different donors. . ΔMFI means delta mean fluorescent intensity.

Supplementary figure 4

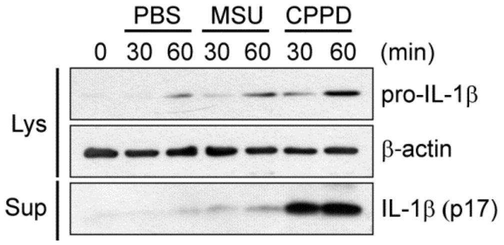

Figure S4. CPPD crystals induce rapid production of pro-IL-1β and secretion of mature IL-1β proteins in primary human monocytes. Human monocytes were stimulated with PBS or MSU crystals (400 µg/ml) or calcium pyrophosphate dehydrate (CPPD) crystals (400 µg/ml) for the indicated time periods. Cell lysates (Lys) were analyzed for the presence of pro-IL-1β and supernatants (Sup) were analyzed for the presence of mature IL-1β (p17) by immunoblot analysis. Data is representative of two independent experiments.

Supplementary figure 5

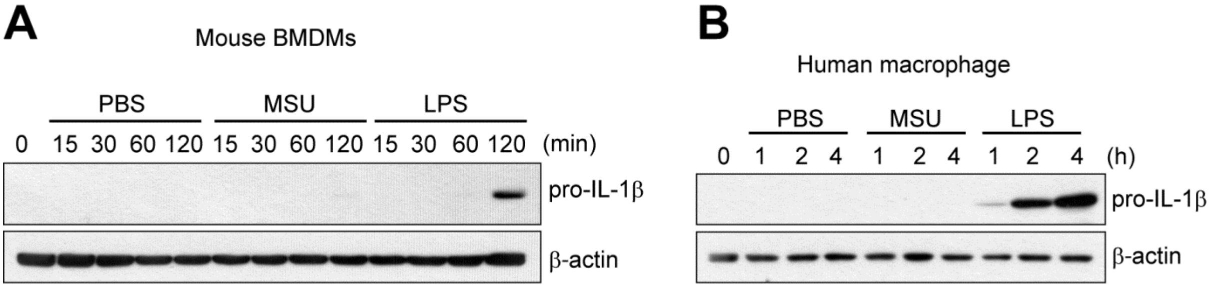

Figure S5. MSU crystal-mediated pro-IL-1β synthesis in monocytes and macrophages is differentially controlled. (A) Mouse bone marrow-derived macrophages (BMDMs) and (B) human monocyte-derived macrophages were stimulated with MSU crystals (400 µg/ml), LPS (100 ng/ml), or PBS as vehicle control for the indicated time periods. Cell lysates were analyzed for the presence of pro-IL-1β by immunoblot analysis. Data is representative of two independent experiments.

Supplementary figure 6

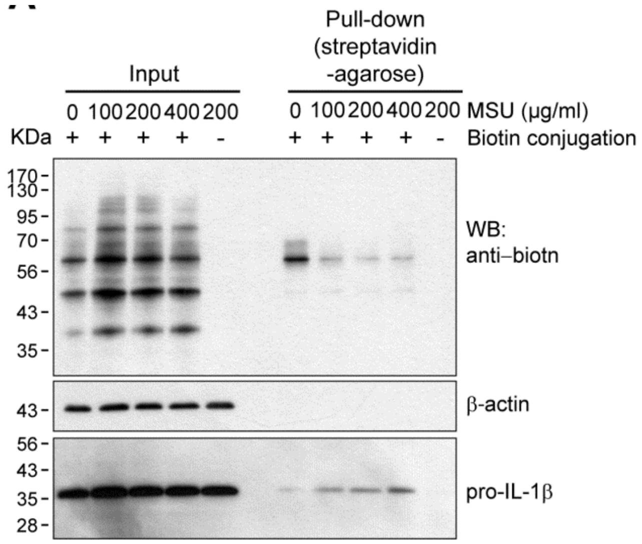

Figure S6. Pro-IL-1β synthesis was enhanced by MSU crystals in a dose-dependent manner. Human monocytes were pre-incubated for 90 min in methionine-free RPMI medium and metabolically labeled for another 60 min with the azide-containing methionine analog AHA (L-azidohomoalaine), followed by treatment with PBS or different concentrations of MSU crystals (100 - 400 μg/ml). Biotin-conjugated newly-synthesized proteins (presented as “Input”) were collected using streptavidin-agarose (presented as “Pull-down”) and the indicated proteins were examined by immunoblot analysis using streptavidin-HRP (upper panel) or anti-IL-1β antibody (lower panel). β-actin was used as an internal control (middle panel). Data is representative of two independent experiments with two different donors.

Supplementary figure 7

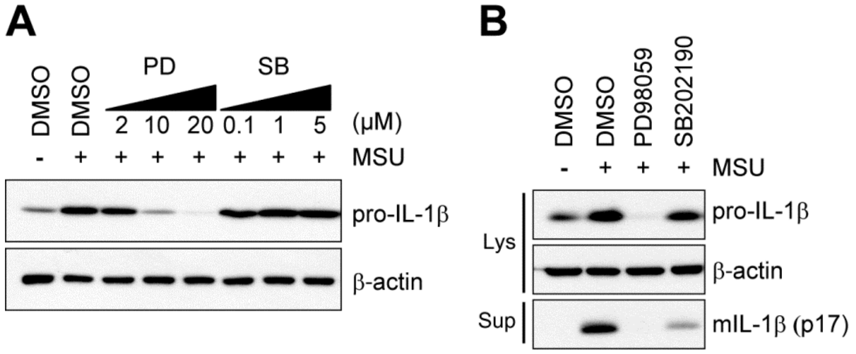

Figure S7. MSU crystal-mediated pro-IL-1β production in THP-1 cells is preferentially regulated via ERK1/2 signaling pathway. THP-1 cells were pre-stimulated for 2 h with 0.5 µM of PMA the day before MSU-treatment. (**A**) THP-1 cells were pre-treated with PD98059 or SB202190 at the indicated concentration for 30 min and then stimulated with MSU crystals (400 µg/ml) for another 5 h. Cell lysates were harvested and pro-IL-1β assessed by immunoblot analysis. β-actin was used as an internal control. (**B**) THP-1 cells were pre-treated with PD98059 (20 µM) or SB202190 (5 µM) for 30 min and then stimulated with MSU crystals (400 µg/ml) for 5 h. Cell lysates (Lys) were analyzed for the presence of pro-IL-1β and supernatants (Sup) were analyzed for the presence of mature IL-1β (mIL-1β; p17) by immunoblot analysis. β-actin was used as an internal control. Data is representative of two independent experiments.

Supplementary figure 8

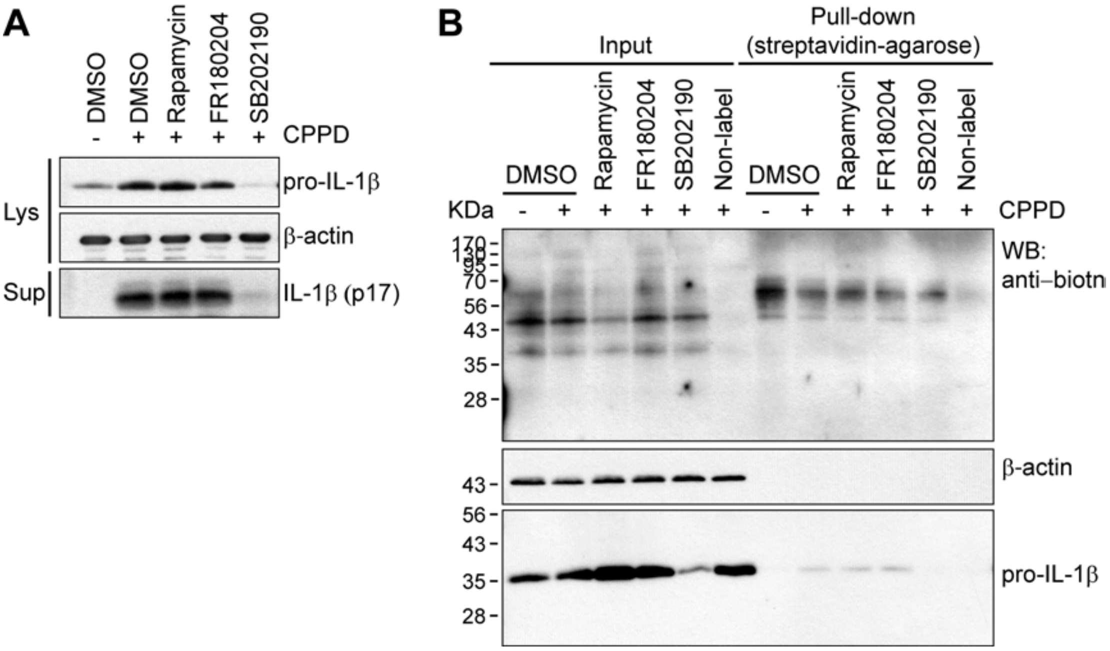

Figure S8. CPPD crystal-stimulated pro-IL-1β protein synthesis is dependent on the p38 MAPK signaling pathway, but not the mTOR and ERK1/2 signaling pathway. Human primary monocytes were stimulated with PBS or calcium pyrophosphate dehydrate (CPPD) crystals (400 µg/ml) in the presence of inhibitors: 50 nM rapamycin, 10 µM FR180204, 5 µM SB202190 and DMSO (vehicle control). Monocytes were pretreated with these inhibitors for 30 min prior to stimulation with CPPD crystals for 60 min. **(A)** Cell lysates (Lys) were analyzed by immunoblot for the effect of the inhibitors on pro-IL-1β synthesis and supernatants (Sup) were analyzed for the presence of mature IL-1β (mIL-1β; p17). Data is representative of two independent experiments. **(B)** Click-iT® Labeling and pull-down assay of biotinylated proteins was performed as previously described in Figure 2. Biotin-conjugated newly-synthesized proteins (Input) were collected by streptavidin-agarose (Pull-down) and the indicated proteins were examined by immunoblot analysis using streptavidin-HRP (upper panel) or anti-IL-1β antibody (lower panel). The non-labeling sample did not have alkyne-derivatized biotin added (negative control). β-actin was used as an internal control in (A and B).
